# New Arsenite Oxidase Gene (*aioA*) PCR Primers for Assessing Arsenite-Oxidizer Diversity in the Environment Using High-Throughput Sequencing

**DOI:** 10.3389/fmicb.2021.691913

**Published:** 2021-10-06

**Authors:** Min Hu, Fangbai Li, Jiangtao Qiao, Chaolei Yuan, Huanyun Yu, Li Zhuang

**Affiliations:** ^1^Guangdong Key Laboratory of Integrated Agro-environmental Pollution Control and Management, Institute of Eco-environmental and Soil Sciences, Guangdong Academy of Sciences, Guangzhou, China; ^2^National-Regional Joint Engineering Research Center for Soil Pollution Control and Remediation in South China, Guangzhou, China; ^3^Ministry of Education Key Laboratory of Pollution Processes and Environmental Criteria, Tianjin Key Laboratory of Environmental Remediation and Pollution Control, College of Environmental Science and Engineering, Nankai University, Tianjin, China; ^4^School of Environment, Jinan University, Guangzhou, China

**Keywords:** *aioA* gene, arsenite oxidation, primer design, As-contaminated paddy soil and sediment, highly-parallel amplicon sequencing

## Abstract

Gene encoding the large subunit of As(III) oxidase (AioA), an important component of the microbial As(III) oxidation system, is a widely used biomarker to characterize As(III)-oxidizing communities in the environment. However, many studies were restricted to a few sequences generated by clone libraries and Sanger sequencing, which may have underestimated the diversity of As(III)-oxidizers in natural environments. In this study, we designed a primer pair, 1109F (5′-ATC TGG GGB AAY RAC AAY TA−3′) and 1548R (5′-TTC ATB GAS GTS AGR TTC AT−3′), targeting gene sequence encoding for the conserved molybdopterin center of the AioA protein, yielding amplicons approximately 450 bp in size that are feasible for highly parallel amplicon sequencing. By utilizing *in silico* analyses and the experimental construction of clone libraries using Sanger sequencing, the specificity and resolution of 1109F/1548R are approximated with two other previously published and commonly used primers, i.e., M1-2F/M3-2R and deg1F/deg1R. With the use of the 1109F/1548R primer pair, the taxonomic composition of the *aioA* genes was similar both according to the Sanger and next-generation sequencing (NGS) platforms. Furthermore, high-throughput amplicon sequencing using the primer pair, 1109F/1548R, successfully identified the well-known As(III)-oxidizers in paddy soils and sediments, and they also revealed the differences in the community structure and composition of As(III)-oxidizers in above two biotopes. The random forest analysis showed that the dissolved As(III) had the highest relative influence on the Chao1 index of the *aioA* genes. These observations demonstrate that the newly designed PCR primers enhanced the ability to detect the diversity of aioA-encoding microorganisms in environments using highly parallel short amplicon sequencing.

## Introduction

Arsenic (As) is a ubiquitous element and an extremely toxic metalloid in the environment. It is either released via natural processes (weathering or hydrothermal and volcanic emissions) or anthropogenic activities (use of arsenical pesticides/herbicides, the release of industrial waste, incineration, and the combustion of fossil fuels) (Oremland and Stolz, 2005). The toxicity and mobility of inorganic As species depend on the redox state. Arsenite [As(III)] is more toxic than arsenate [As(V)] to an active cell. As(V) is much more strongly adsorbed by commonly occurring surface minerals, such as aluminum oxyhydroxides or iron (hydro)oxides, than As(III) (Oremland and Stolz, 2005).

As(III) oxidizing microorganisms exist widely in nature as both heterotrophic and chemo/photosynthetic autotrophic microorganisms (Hamamura et al., 2009). As(III) oxidation in nature is primarily mediated by microorganisms and catalyzed by the Aio enzyme system. The Aio enzyme contains two subunits: a large catalytic subunit (AioA, approximately 90 kDa) that contains a molybdopterin center bound to the pyranopterin cofactor and a [3Fe–4S] cluster and an associated small subunit (AioB, approximately 14 kDa) containing a single Rieske-type [2Fe–2S] cluster (Mukhopadhyay et al., 2002). Homologous genes encoding these two subunits were formerly assigned different names (*aoxB* and *aoxA*, *aroA* and *aroB*, and *asoA* and *asoB*, accordingly). The nomenclature for genes involved in prokaryotic As(III) oxidation has been unified as the name *aio*, and the two subunits are now denoted as *aioA* and *aioB*, respectively (Yamamura and Amachi, 2014). To date, homologs of genes encoding AioAB have been found in phylogenetically diverse strains including members of α-,β-,γ-*Proteobacteria*, *Actinobacteria*, *Aquificae*, *Bacteroidetes*, *Chlorobi*, *Chloroflexi*, *Crenarchaeota*, *Deinococcus-Thermus*, *Firmicutes*, and *Nitrospira* (Yamamura and Amachi, 2014). The *aioA* gene has been successfully used as a molecular marker to capture the diversity and taxonomic information for As(III)-oxidizing microbial communities in environments (Zhang et al., 2017; Xue et al., 2020; Zhai et al., 2020). Primers targeting the *aioA* genes, such as M1-2F/M3-2R (approximate amplicon size of 1,100 bp) (Quéméneur et al., 2008) and deg1F/deg1R (530 bp) (Inskeep et al., 2007) have been developed. The *aioA* gene amplicon size obtained using some recently reported primer pairs ranged from 530 to 1,100 bp (Inskeep et al., 2007; Quéméneur et al., 2008; Hamamura et al., 2009; Sultana et al., 2012), which are not feasible for application in highly parallel amplicon sequencing for which an amplicon size of less than 500 bp is recommended. However, classical surveys of *aioA* diversity are performed by constructing clone libraries and Sanger sequencing, which have limitations of the sample number and sequencing depth (normally dozens of clones per sample), which possibly underestimate the *aioA* diversity in natural environments.

Simultaneous comparative analysis of the bacterial community structure across many samples is currently made possible by the advent of next-generation sequencing (NGS) technologies for highly multiplexed amplicon sequencing of 16S rRNA genes (Klindworth et al., 2012). Amplicon NGS surveys are now also increasingly applied to target functional genes that are indicative of a particular microbial guild, such as *mcrA* for methane oxidizers (Cai et al., 2018), *amoA* for ammonia oxidizers (Roots et al., 2019), *pmoA* for methanotrophs (Chiri et al., 2020), and *dsrAB* for sulfate reducers (Zeleke et al., 2013; Pelikan et al., 2016). To date, investigations targeting metal biotransformation using NGS have only involved the *arrA* gene for dissimilatory arsenate reducers (Mirza et al., 2017) and the *arsM* gene for arsenate methylated microorganisms (Zhang S. Y. et al., 2015). However, no available PCR primer is suitable for applications in high-throughput sequencing for genes that encode As(III)-oxidases.

The aim of the current study is to design degenerate primers that target the *aioA* gene and have the subsequent amplicon length suitable for highly multiplexed amplicon sequencing by NGS. The specificity and coverage of the newly designed primers are then compared with the primer sets previously published *in silico* and experimentally by constructing clone libraries. Finally, the utility of the newly designed primer pair for highly parallel amplicon sequencing is verified with As-contaminated paddy soil and sediment samples using the Illumina Hiseq platform. The newly designed primer pair constitutes a useful tool for the further study of microbial communities to investigate the diversity of As(III) oxidizers in environmental samples.

## Materials and Methods

### Design and Evaluation of the *aioA*-Targeting Primers

A total of 67 full-length bacterial AioA proteins (orthology number: K08355) and *aioA* gene sequences were downloaded from the KEGG database (Kanehisa and Goto, 2000) (version 91.0, released on July 1, 2019) (Supplementary Table 1). The taxonomic profiles of these AioA sequences were obtained using a Perl script utilizing a tax dump file from the NCBI FTP (ftp://ftp.ncbi.nlm.nih.gov/pub/taxonomy/). *Proteobacteria*-related sequences were in the greatest abundance (accounting for 87.67% of all sequences), followed by *Deinococcus-Thermus* (5.48%). At the order level, the AioA sequences originated from *Burkholderiales* (39.73%) and *Rhizobiales* (20.55%), which were predominant, followed by *Rhodobacterales* (10.96%) and *Thermales* (5.48%) (Supplementary Figure 1). Phylogenetic analysis of the AioA sequences was conducted using MEGA version 7 (Kumar et al., 2018). Their evolutionary history was inferred using the Neighbor-Joining method and bootstrapping of 1,000. All of the positions containing gaps and missing data were eliminated. The *aioA* gene sequences, all with their complete coding regions, were aligned using MUSCLE software v3.8.31 (Edgar, 2004) with the default parameters. Then, the aligned sequences encoding the conserved molybdopterin center and the [3Fe-4S] binding motif of the *aioA* protein were extracted and used for the downstream primer designs. These two alignments were then imported into DegePrime (Hugerth et al., 2014) to generate degenerate primers for conserved regions unique to the *aioA* gene at the following parameters: oligomer length, 20; maximum oligomer degeneracy, 96; the number of bases at the sequence termini that would not be considered in the analysis, 30. Only the sequence alignments of the conserved molybdopterin center of the AioA successfully produced primers using DegePrimer were namely 1109F (5′-ATC TGG GGB AAY RAC AAY TA−3′) and 1548R (5′-TTC ATB GAS GTS AGR TTC AT−3′) (corresponding to the nucleotides 1,168–1,184 and 1,628–1,609 of the *aioA* gene of *Alcaligenes (A.) faecalis*) (Table 1). The 1109F/1548R primer sets were *in silico* predicted and generated a PCR product size of approximately 450 bp, and these are suitable for parallel amplicon sequencing using NGS platforms such as Illumina Hiseq PE250 or the ThermoFisher Ion S5 XL system.

**TABLE 1:** Primers for the amplification of the *aioA* genes.

Primer set	Sequences (5′–3′)	Primer Deg	Tm (^*o*^C)^*a*^	Sequences used for design	Hit^*b*^	Amplicon size (bp)	Corresponding position on CDS			References
							*A. faecalis* NCIB 8687	*A. tume faciens*	Rhizobium sp. NT-26	
1109F	ATCTGGGGBAAYRACAAYTA	24	54–66	67	68.40%	439	1,168–1,184	1,246–1,262	1,246–1,265	This study
1548R	TTCATBGASGTSAGRTTCAT	24	58–63				1,628–1,609	1,715–1,696	1,715–1,696	
M1-2F	CACTTCTGCATCGTGGG NTGYGGNTA	32	71–78	9	46.10%	1,100	70–86	73–95	73–95	Quéméneur et al., 2008
M3-2R	TGTCGTTGCCCCAGATGADN CCYTTYTC	48	71–78				1,180–1,168	1,261–1234	1,261–1,234	
deg1F	GTSGGBTGYGGMTAYCA BGYCTA	288	64–79	7	75.00%	530	79–95	91–107	91–107	Inskeep et al., 2007
deg1R	TTGTASGCBGGNCGRTTRT GRAT	192	63–76				605–589	611–595	614–598	

*^*a*^The Tm values were calculated on the NEB Tm Calculator (http://tmcalculator.neb.com/#!/main), and different Tm values originated from primer degeneracies.*

*^*b*^A hit was considered when the forward and reverse primers both matched the same sequence in the 67 aioA dataset with a maximum of two mismatches and an alignment length greater than 16 nt.*

Then the performance of the newly designed primer set was compared with the other two previously published and commonly used primers, i.e., M1-2F/M3-2R (Inskeep et al., 2007) and deg1F/deg1R (Quéméneur et al., 2008). Direct amplification of the *aioA* genes from pure culture isolates were not evaluated because the isolates were not available in our laboratory. An *in silico* evaluation of the primers against the 67 *aioA* gene sequences from the KEGG database using the BLASTN algorithm was performed. A hit was considered when the forward and reverse primers both matched the same sequence, with a maximum of two mismatches and an alignment length larger than 16 nt. Coverage was displayed as the ratio of sequence hits in all the constructed 67 *aioA* gene dataset. Furthermore, a taxonomic analysis of the hit *aioA* gene sequences was also performed to estimate the phylogenetic coverage of each primer pair.

### Comparison of the 1109F/1548R Primers and the Commonly Reported Ones Using Clone Libraries

As-contaminated paddy soil and sediment samples were selected to estimate the amplification efficiency and performance of the newly designed primer pair, 1109F/1548R, together with another two published *aioA*-targeting primer pairs (M1-2F/M3-2R and deg1F/1R). The soil sample was collected from an As-contaminated paddy field (23°37′28″′N, 116°48′43″E) that was surrounded by the Lianhuashan tungsten mine located in Shantou City, Guangdong Province, China. The severe impact of mining and metal processing activities on the paddy field has been documented (Liu et al., 2010, 2015). Soil samples were randomly collected from the arable layer (0–20 cm deep) and combined into one compost sample in 50-ml polypropylene centrifuge tubes. The sediment sample was collected from the river nearby the As-contaminated paddy soil field. Samples were then transported on ice to the laboratory and stored at −80°C prior to DNA extraction. For the soil physico-chemical properties analysis, the soil samples were air-dried at room temperature and crushed using a wooden roller to pass through a 2 mm sieve. The soil was characterized, and the results were a total organic C content of 35.12 g kg^–1^, total N of 2.72 g kg^–1^, total P of 0.86 g kg^–1^, total K of 18.2 mg kg^–1^, and total As of 102.5 mg kg^–1^, with a pH of 5.75. The physico-chemical properties of the sediment are shown in Supplementary Table 1 (listed as S1 sample). Genomic DNA from the 0.5 g paddy soil and sediment samples was extracted using the PowerSoil^TM^ DNA isolation kit (MoBio, Carlsbad, CA). All extractions were performed in triplicate according to the manufacturer’s instructions, and the extracted DNA was stored at −80°C until analysis.

The DNA extracted from the paddy soil and sediment was amplified using the 1109F/1548R primer pair and the two published primer pairs, M1-2F/M3-2R and deg1F/deg1R. Each PCR reaction contained 25 μl of a PCR mixture consisting of 5 μL 5 × PCR *ExTaq* buffer, 100 μmol dNTPs, 0.5 μmol each primer, 50 ng template, and 1 U of *ExTaq* DNA polymerase (TaKaRa, Dalian, China). Following verification of the amplification reaction on a 1.5% agarose gel, the PCR product was excised and purified using a QIAquick gel extraction kit (Qiagen, Valencia, CA). The purified PCR products were cloned into a pGEM-T vector (Promega, Madison, WI) and used to transform *Escherichia coli* DH5α competent cells (TaKaRa, Dalian, China) according to the protocol provided by the manufacturer. Approximately 100 clones from each library were sequenced using the ABI Prism 3730 Genetic Analyzer (Applied Biosystems, Foster City, CA). The cloned sequences were trimmed and assembled using SeqMan (DNAstar, Madison, WI) after verification using a BLASTX similarity search against the NCBI-nr database at e < e^–10^ and an alignment length > 150 nt. The specificity of the primer sets was calculated using the *aioA*-annotated sequence against the total number of cloned sequences. The BLASTX analysis was also used for the taxonomic assignment of the *aioA* sequences.

### Evaluation of the 1109F/1548R Primer Pairs Using High-Throughput Amplicon Sequencing

Samples from the As-contaminated paddy soil and sediment were collected from the region surrounding the Lianhuashan tungsten mine, as described elsewhere (Liu et al., 2010, 2015; Supplementary Table 2). Arsenic species (including dissolved, phosphate-extractable and oxalate-extractable As(III) and As(V)) were detected as described (Yu et al., 2017; Qiao et al., 2019). In brief, the dissolved As was determined by adding 20 ml of ultrapure water (pH = 7.0) to 1 g of soil and shaking in a rotary shaker (200 rpm) for 15 m at room temperature. This was followed by centrifugal separation at 4,500 r/min for 15 m. The supernatant then was then filtered through a sterile 0.22-μm filter for determination of the dissolved As(III) and As(V) concentrations using atomic fluorescence spectroscopy (SA-20, Jitian, Beijing, China). Soil pellets were used to sequentially fractionate arsenic in the soil using 1M KH_2_PO_4_ + 0.1 M ascorbic acid and 0.2 M NH_4_^+^-oxalate that primarily target As adsorbed on iron (oxyhydr)oxides and As incorporated into iron (oxyhydr)-oxides, respectively. The mixtures were shaken and centrifuged. Then, each supernatant was passed through a 0.22 μm filter, and the As concentrations were determined using atomic fluorescence spectroscopy as described above.

For the parallel amplicon sequencing of the *aioA* gene, DNA from the As-contaminated paddy soil and sediment was extracted using the PowerSoil^TM^ DNA isolation kit (MoBio) and amplified using the 1109F/1548R primer pair with Illumina adapters and a unique 12bp-Golay barcode sequence attached to the reverse PCR primer (Supplementary Table 3). The PCR conditions were as follows: 3 m at 95°C; followed by 28 cycles of denaturation at 95°C for 30 s, primer annealing at 55°C for 30 s, an extension at 72°C for 30 s, and a final extension for 5 m at 72°C. The PCR products were checked by resolving an aliquot on a 2% agarose gel. Triplicate reactions were combined and the amplicons were purified using a QIAquick gel extraction kit (Qiagen). The concentration of purified PCR products was determined by using a Qubit 3.0 Fluorometer and the Qubit dsDNA BR Assay kit (Life Technologies, Grand Island, NY) following the manufacturer’s instructions. The purified PCR products were pooled in equimolar concentrations and sequenced using an Illumina Hiseq platform at Novogene Bioinformatics Technology (Beijing, China). The HiSeq analysis was performed in the 2 × 250 cycle combination, i.e., producing 2 × 250 nt paired-end reads.

The paired-end reads were merged using Flash (Magoč and Salzberg, 2011), followed by quality control using the QIIME pipeline (Caporaso et al., 2010b). The quality control included retaining sequences with minimum merged sequence lengths of 250 bp and maximum homopolymers of eight bases. In addition, sequences with unidentified bases (N) and sequences with more than one inexact match with a unique barcode identifier and perfect primer matches were also removed. The remaining sequences were aligned using PyNAST (Caporaso et al., 2010a) and then uploaded into the UPARSE pipeline implementing USEARCH (Edgar, 2010) for the dereplication and *de novo* clustering to a 90% similarity level after the removal of singletons and chimeras. Denoising was performed using the PyroClean software (Ramirez-Gonzalez et al., 2013) with default parameters. The conservation in the sequences of gene candidates in element biogeochemical cycles is much lower than that of 16S rRNA and ITS genes, which are phylogenetic markers used for microbial community analyses. The standard threshold of operational taxonomic unit (OTU) clustering has not been unified for arsenic metabolism genes. In this study, the *aioA* sequences were clustered into OTUs at a 90% identity threshold. Representative OTU sequences were then confirmed as the *aioA* gene sequences using the Diamond software (Buchfink et al., 2014) against the NCBI-nr database at e < e^–10^ and an alignment length > 200 nt. Phylogenetic trees were generated using FastTree and representative OTU sequences (Price et al., 2010). To assess the OTU abundance, UPARSE was used to map the *aioA* sequences to the representative OTU sequences at a 90% identity threshold (as a cutoff of the subgenus level). A distance matrix was then constructed based on the OTU abundance. To estimate the alpha diversity, a random subsampling method for each sequence library was used for the microbial community diversity index calculations to control for the effects of the library size. The alpha diversity indices (observed OTUs, Chao1, phylogenic diversity [PD], Shannon, and Simpson) were calculated for all the samples with 1,000 repetitions using a size of 33,637 sequences per sample. Furthermore, the beta (unweighted Unifrac) diversity was calculated in QIIME software (Caporaso et al., 2010b). The taxonomic composition of the *aioA* sequences in each sample was determined using the MEGAN software (Huson et al., 2007), with the results searched against the NCBI-nr using Diamond software (Buchfink et al., 2014). The phylogeny of the abundant representative OTU sequences whose average relative abundance exceeded 1% was determined using MEGAX (Kumar et al., 2018).

### Statistics

Statistical analyses were conducted using SPSS 17.0 (SPSS Inc.). Statistical significance was estimated using two-way analyses of variance (ANOVA) to determine the significance in the differences of alpha diversity between the paddy soil and the sediment samples. The Spearman’s correlation coefficient (rho) was used as a measure of the correlation between the taxonomic compositions of *aioA* genes sequenced by Sanger and NGS platforms, giving a value between + 1 and -1, where 1 stands for the total positive correlation, 0 stands for no correlation, and –1 stands for the total negative correlation. The random forest (RF) analysis was conducted using the R package randomForest to investigate the impact of environmental parameters on the *aioA* gene diversity (Liaw and Wiener, 2002).

### Accession Numbers

The amplicon sequences of the *aioA* gene from paddy soil and sediment samples have been deposited in the NCBI Sequence Read Archive (SRA) under accession number SRR9915017-SRR9915024.

## Results

### Evaluation of the Phylogenetic Coverage and Specificity of 1109F/1548R and Two Published Primers

The specificity of 1109F/1548R together with another two previously published *aioA*-targeted primers, M1-2F/M3-2R and deg1F/deg1R (Table 1), was compared to the 67 *aioA* gene sequences from the KEGG database. The sequences matched by the three primer pairs primarily originated from *Proteobacteria*, especially from the orders of *Burkholderiales*, *Rhizobiales*, and *Rhodobacterales* (Supplementary Figure 1). Primer pairs 1109F/1548R exactly matched the *aioA* gene sequences from the well-studied As(III)-oxidizers, such as *A. faecalis* subsp. *faecalis* NCIB 8687, *Rhizobium* sp. NT-26, *Xanthobacter autotrophicus* Py2, *Achromobacter xylosoxidans* NCTC10807, *Herminiimonas arsenicoxydans*, *Thiomonas arsenitoxydans*, and *Chloroflexus aurantiacus*. The phylogenetic coverage of the different primer pairs was different. Primer pairs 1109F/1548R, M1-2F/M3-2R, and deg1F/deg1R targeted sequences from 31, 31, and 40 genera, respectively (the 67 *aioA* sequences used for the primer design covered 43 genera) (Supplementary Figure 1). No primer pairs tested in the current study matched all the *aioA* gene sequences retrieved from the KEGG database.

**FIGURE 1:**
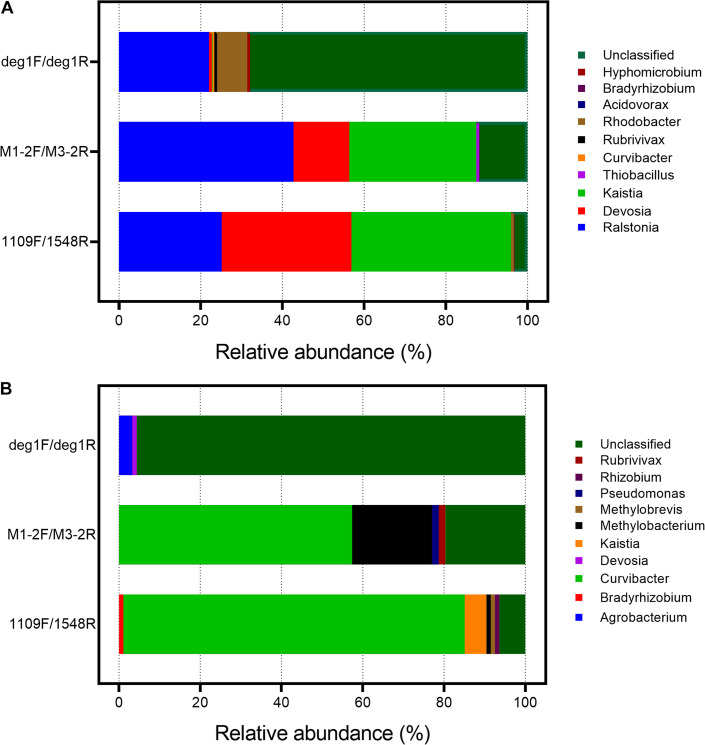
Taxonomic information of the clone sequences generated using three primer pairs and DNA from the As-contaminated paddy soil **(A)** and sediment **(B)**. The *aioA*-targeted clone sequences were verified using the BLASTX similarity search against the NCBI-nr database at e < e^– 10^ and alignment length > 150 nt. Specificities of the primer sets were calculated as the *aioA*-target sequence hits to the total number of clone sequences. The taxonomic information for the *aioA* clone sequences is shown at the genus level after disarming non-*aioA* sequences.

The phylogenetic distribution of the amplified sequences and specificity of the primer sets were compared by constructing clone libraries using DNA isolated from As-contaminated paddy soil and sediment samples (100 clones for each primer pair). The taxonomic information for the *aioA* gene sequences in the three clone libraries was also compared. At the genus level, most of the *aioA* sequences from the deg1F/deg1R libraries were unclassified both in the paddy soil and sediment samples, with relative abundances of 68.61 and 99.65%, respectively (Figure 1). For the paddy soil sample, the 1109F/1548R library data were similar to the deg1F/deg1R data in that *Ralstonia* sequences accounting for 25.16 and 22.03%, respectively, of all the *aioA* sequences compared with 42.72% using the M1-2F/M3-2R primer pair. However, *Devosia* and *Kaistia* predominantly been identified using the 1109F/1548R and M1-2F/M3-2R primer pair (Figure 1A). A sediment sample was also selected to compare the differences in the taxonomic distribution among these three *aioA* gene primer pairs (Figure 1B). The *Curvibacter*-associated *aioA* gene sequences consisted of 57.38 and 84.04% in the M1-2F/M3-2R and 1109F/1548R libraries, respectively, and was a rare biome in the deg1F/deg1R library. Furthermore, the dominant *Methylobacterium*-affiliated sequence (19.67%) has only been detected using the M1-2F/M3-2R primer pair (Figure 1B).

### A Comparison of 1109F/1548R on the Sanger and NGS Sequencing Platforms

**FIGURE 2:**
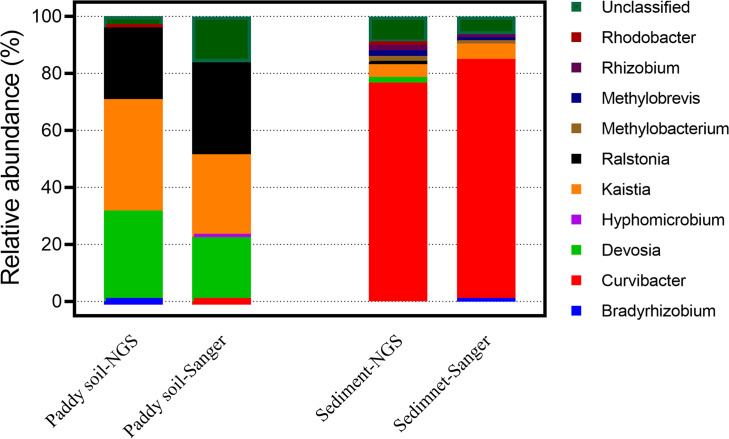
The taxonomic comparison of Miseq (NGS) and clone (Sanger) sequencing of *aioA* genes in paddy soil and sediment samples using 1109F/1548R primers.

A comparison of 1109F/1548R on the Sanger and NGS sequencing platforms was also conducted to verify the effects of sequencing methods on the detected community of As(III)-oxidizing bacteria using the 1109F/1548R primer pair. It was shown that the taxonomic compositions of the *aioA* genes were similar using these two sequencing strategies. Although, the taxonomic composition of the *aioA* genes had slight differences between the Sanger and NGS sequencing, the most abundant *aioA* genes from the paddy soil sample were affiliated with *Kaisita* (with a relative abundance of 28.94% in the Sanger sequencing and 39.05% in the NGS sequencing). This was followed by *Devosia* (Sanger: 22.53%; NGS: 31.77%) and *Ralstonia* (Sanger: 32.30%; NGS: 25.16%). The predominant genera in the sediment examined using the 1109F/1548R primer pair were also similar both on the Sanger and NGS platforms, and *Curvibacter* (Sanger: 84.04%; NGS: 76.80%) was the most identified abundant genera (Figure 2). Spearman’s rho tests showed a significant correlation between taxonomic compositions of *aioA* genes sequenced by Sanger and NGS platforms, using paddy soil (Spearman rho coefficient = 0.838, *p*-value = 0.009) and sediment (Spearman rho coefficient = 0.829, *p*-value = 0.002) samples. The above results suggested the taxonomic profiles are similar between these two sequencing approaches.

### Diversity and Composition of the *aioA* Genes in the Paddy Soil and Sediment Samples

**FIGURE 3:**
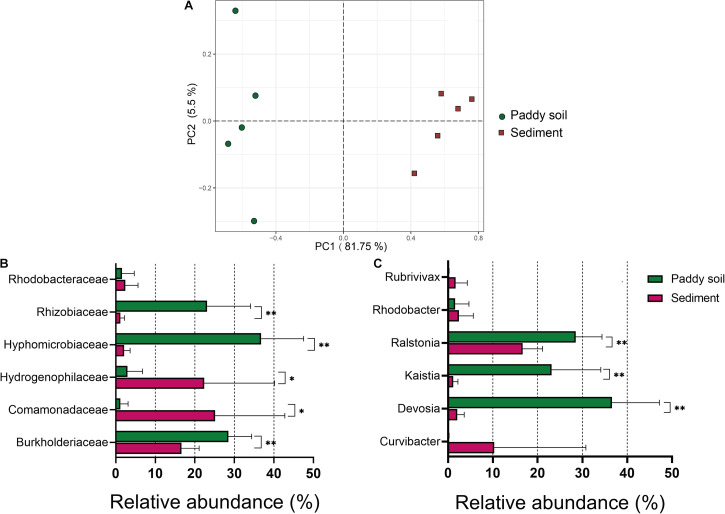
Differences in the As(III)-oxidizing bacteria between the paddy soil and sediment samples. **(A)** Unweighted PCoA clustering of the As(III)-oxidizing community in the paddy soil (red circles) and sediment (blue squares) samples generated using the 1109F/1548R primer sets. The percentage variation in the plotted principal component is indicated on the axes. **(B)** Taxonomic composition of the As(III)-oxidizing bacteria at the family level. **(C)** Taxonomic composition of the As(III)-oxidizing bacteria at the genus level. The taxonomic composition of the *aioA* sequences in each sample was determined using the MEGAN software against the NCBI-nr with Diamond software at e < e–10 and an alignment length > 200 nt. The average ± SD values of the samples in each group are expressed in each column. The significance cutoff for the corrected *P*-value using the Benjamini–Hochberg false discovery rate (FDR) procedure is shown. Significant differences between the sampled groups are indicated as ^∗∗^*p* < 0.01 and ^∗^*p* < 0.05.

The availability of the newly developed primer pair, 1109F/1548R, for the amplification of *aioA* genes from five samples of As-contaminated paddy soil and five samples of sediment was tested using high-throughput sequencing (Supplementary Table 4). In addition, the community diversity and structure of the As(III) oxidizers from these two habitats were compared. After quality screening, chimera removal, and the BLASTX analysis against the NCBI-nr database, 151,287 *aioA* sequences were extracted from the 1109F/1548R libraries (Supplementary Table 4). After resampling, 10,313 sequences per sample were used for further analysis. The *aioA* amplicon sequences were clustered into 280 OTUs at a 90% sequence identity. The Good coverage ranged from 0.996 to 0.998, indicating that the sequencing had sampled the majority of the diversity present (Supplementary Table 4). The alpha diversity of *aioA* genes, as measured by the observed OTUs, Chao1, Shannon, Simpson, and Faith PD indexes, was estimated using the QIIME2 platform. Briefly, the observed OTUs were 102.6 ± 18.022 and 81.4 ± 21.22 for the paddy soil and sediment samples, respectively. The Chao1 indexes were 128.97 ± 19.21 and 104.64 ± 22.39 for the paddy soil and sediment samples, respectively. The Shannon indexes were 3.47 ± 0.61 and 3.20 ± 0.47 for the paddy soil and sediment samples, respectively. The Simpson indexes were 0.81 ± 0.07 and 0.79 ± 0.06 for the paddy soil and sediment samples, respectively. The Faith PD indices were 12.63 ± 1.36 and 10.90 ± 1.64 for the paddy soil and sediment samples, respectively. The Chao1 and Faith PD diversity indices were significantly higher in the paddy soil than in the sediment (both *p* = 0.076, Supplementary Table 5). However, the number of observed OTUs and the values of the Chao1, Shannon, and Simpson indexes did not differ significantly between the paddy soil and sediment samples (all *p* > 0.05, Supplementary Table 5). The results of the unweighted Unifrac PCoA showed that the sediment samples clearly clustered together and separately from the paddy soil samples, and the Unifrac distance among the paddy soil samples was higher than that among the sediment samples. These results indicated that the As(III)-oxidizing community structure was strongly associated with the sampled habitats (Figure 3A).

The phylogenetic distribution of the As(III)-oxidizing communities was also determined using the BLASTX data. Among the *aioA* sequences retrieved from the paddy soil, the largest fraction of these sequences was affiliated with the families *Hyphomicrobiaceae* (36.79 ± 10.75%), *Rhizobiaceae* (23.14 ± 10.97%), and *Burkholderiaceae* (28.50 ± 5.88%) (Figure 3B). Only 2.96 ± 3.84%, 1.60 ± 3.07%, and 1.19 ± 1.94% of the sequences *aioA* were distributed among the families *Hydrogenophilaceae*, *Rhodobacteraceae*, and *Comamonadaceae*, respectively. For the As-contaminated sediments, the average, 25.16 ± 17.6% sequences fell into the family *Comamonadaceae*, and 22.39 ± 17.78% was affiliated with *Hydrogenophilaceae*, followed by *Burkholderiaceae* (16.65 ± 4.45%). Multiple *t*-test analyses indicated significant differences in the abundance (*p* < 0.05) between the dominant families in the paddy soils and the sediments (with relative abundances greater than 1% including *Burkholderiaceae*, *Comamonadaceae*, *Hydrogenophilaceae*, *Hyphomicrobiaceae*, and *Rhizobiaceae*) (Figure 3B). At the genus level, *Ralstonia*, *Kaistia*, and *Devosia* exhibited statistically significant differences in abundances between the paddy soil and the sediment samples (*P* < 0.01) (Figure 3C).

To investigate the phylogenetic affiliation of the most abundant OTUs in detail, the 20 most abundant OTUs were aligned with reference sequences and a Neighbor-Joining tree was constructed. As shown in Supplementary Figure 2 and Supplementary Table 6, the majority of the abundant OTUs clustered with uncultured bacteria. The 20 most abundant OTUs were grouped into seven clades that belonged to *Alphaproteobacteria* (in the orders of *Hyphomicrobiales* and *Rhodobacterales*), *Betaproteobacteria* (in the orders of *Burkholderiales* and *Hydrogenophilales*), and uncultured bacteria (three clusters). Clade I consisted of two OTU (OTU 11 and OTU 106) and showed high similarity with bacterium HR39 and bacterium HR40) with additive abundances of 23.65 and 1.12% in the paddy soil and sediment samples, respectively. In addition, two OTUs (OTU 13 and OTU 170 with total abundances of 33.15 and 1.53% in the paddy soil and sediment samples, respectively) clustered with *Devosia* sp. 67–54 (OJX19890) (with an average 82.75% amino acid identity). Furthermore, OTU 40 (average abundance in the paddy soil: 1.57%; sediment: 2.36%) clustered closely with *Rhodobacter* sp. CACIA14H1 (ESW59939) (93.00% identity). The abundance of OTUs (OTU3 and OTU 162) of clade III in the paddy soil (with total abundances of 1.00%) was lower than that in the sediment (25.79%), which clustered with *Curvibacter* sp. GWA2 64 110 (OGP03110) (89.44% identity). Three OTUs (OTU 1, OTU 42, and OTU 16, with total abundances of 1.18 and 20.15% in the paddy soil and sediment samples, respectively) clustered with sequences from a reference *Hydrogenophilaceae* bacterium CG1_02_62_3903 (OIO79863) (with an average 79.58% amino acid identity). Six OTUs (OTU 20, OTU 258, OTU 105, OTU 251, OTU 38, and OTU 12, and with total abundances of 16.39 and 8.75% in the paddy soil and sediment samples, respectively) and with an uncultured microorganism (BAM75656) (average 76.21% amino acid identity). Finally, OTU 2, OTU 5, OTU 32, and OTU 68 (with a total abundance in the paddy soil: 1.7%; sediment: 13.93%) clustered with *Betaproteobacteria* bacterium RIFCSPLOWO2_02_FULL_62_17 (OFZ99153) (average 86.64% identity). The remaining OTUs were placed closely together with a number of uncultured *aioA* sequences obtained in previous studies that were not affiliated with any cultured isolate (Supplementary Figure 2 and Supplementary Table 6).

### Correlation Between the *aioA* Gene Diversity and Geochemical Variables

**FIGURE 4:**
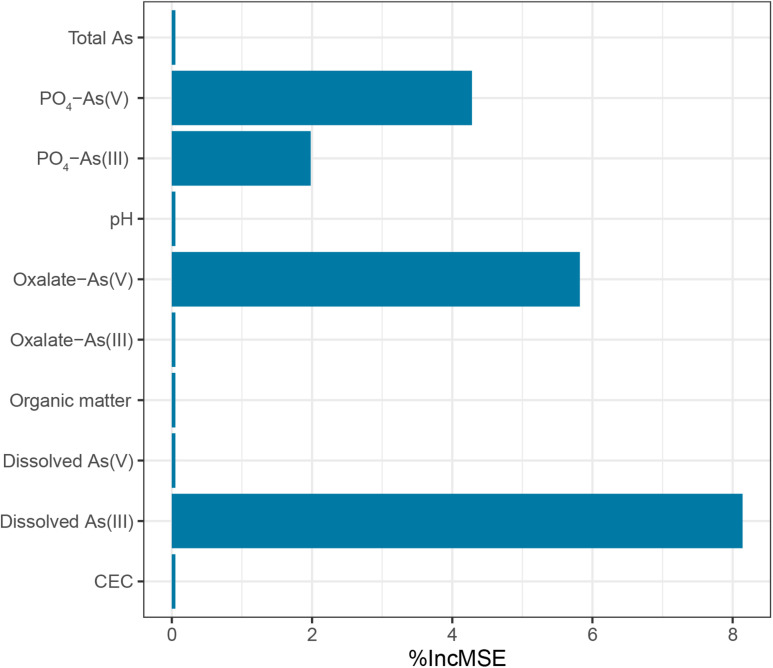
Random forest (RF) predictions of the most influential environmental parameters on the Chao1 index of the *aioA* genes.

Samples from the As-contaminated paddy soil and sediment were collected from the same region that was used to examine the performance of the 109F/1548R primer pairs using high-throughput amplicon sequencing. The paddy samples exhibited a pH range from 6.44 to 6.75 compared to a range of 6.15–6.49 in the sediment samples. The pH values in the paddy soil samples were significantly higher than that in the sediment samples (Student’s *t*-tests, *p* < 0.01). The paddy soil and sediment samples contained organic matter concentrations of 27.86 ± 2.50 g kg^–1^ and 27.11 ± 8.55 g kg^–1^, respectively. The total As concentration in the paddy soil samples varied from 37.19 to 94.42 mg kg^–1^, and all exceeded the maximum allowable concentration (MAC) of As for agricultural soils in China (30mg kg^–1^, National Environmental Protection Agency of China GB 15618, 1995). The total concentration of As in the sediment samples was 57.50 ± 21.30 mg kg^–1^, which did not exhibit a significant difference to the paddy soil samples. Phosphate-extractable and oxalate-extractable As(III)/As(V) in the paddy soil and sediment samples, which represented the As species adsorbed on iron (oxyhydr) oxides and the As species incorporated into iron (oxyhydr)-oxides, respectively. The concentration of phosphate-extractable As(III)/As(V) and oxalate-extractable As(III)/As(V) in the sediment samples (PO_4_- As(III) with an average ± SD value of 4.13 ± 4.00 mg kg^–1^; PO_4_- As(V) value of 20.34 ± 13.67 mg kg^–1^; oxalate-As(III) value of 1.26 ± 0.68 mg kg^–1^; and oxalate-As(V) value of 9.84 ± 4.78 mg kg^–1^) were significantly higher than that in the paddy soil sample (PO_4_-As(III) value of 1.30 ± 0.95 mg kg^–1^; PO_4_-As(V) value of 6.08 ± 2.93 mg kg^–1^; oxalate-As(III) value of 0.75 ± 0.22 mg kg^–1^; and oxalate-As(V) value of 3.52 ± 0.86 mg kg-1) (all *p* < 0.01). RF predictions were employed to explore how the environmental parameters affected the diversity of *aioA* (Figure 4). The results showed that dissolved As(III) had the highest relative influence (8.14%) on the Chao1 index of the *aioA* genes followed by oxalate-As(V) (5.82%), PO_4_-As(V) (4.28%), and PO_4_-As(III) (1.98%). These findings implied that dissolved As(III) may be a key factor driving the species richness of As(III)-oxidizing bacteria in natural environments.

## Discussion

### Comparative Performances of Various *aioA* Gene Primer Pairs

Based on the *in silico* evaluation, the best performing primer pair was deg1F/deg1R, with an overall coverage of 85.09% of all the *aioA* gene sequences analyzed (Supplementary Figure 1). That was because of primer degeneracy of deg1F and deg1R was much higher than that of the other two primer pairs (Table 1). The high degeneracy of primers increased the probability of matching *aioA* gene sequences. However, it should be noted that primer degeneracy can also lead to a biased picture of microbial diversity (Baker et al., 2003; Huse et al., 2008), and preferential amplification might lead to under-representation of important members of a community (Huse et al., 2008). In addition, the primers used for high-throughput amplicon sequencing normally consisted of the Illumina adapter, barcoder, pad, and linker sequences, followed by the gene targeted primers (Caporaso et al., 2010b). Then, the high degeneracy of the *aioA* primers may also increase the probability of secondary structure generation, such as dimers and hairpins. Such a secondary structure of the primers adversely affect primer template annealing and reduce the availability of primers to participate in the reaction. This can lead to poor or no yields of the product. Thus, the high degeneracy of the *aioA* primers are not suitable for high-throughput amplicon sequencing.

Although the phylogenetic coverage of deg1F/deg1R was best in the *in silico* analysis, the taxonomic resolution was not high in the other reports that used this primer pair. In an investigation of the impact of nitrate additions on As species dynamics in the porewater of four As-contaminated paddy soils, the results of the *aioA* clone libraries using deg1F/deg1R showed that 35.42–70.83% of the clone sequences were classified as uncultured organisms (Zhang et al., 2017). In a similar study on the diversity of bacterial As(III) oxidase and reductase genes in the rhizosphere of the As-hyperaccumulator, the result of the *aioA* gene clone libraries using the deg1F/deg1R primer pair also showed that most of the clone sequences were affiliated with uncultured bacterium (Han et al., 2017). Hence, the phylogenetic coverage of deg1F/deg1R should be carefully examined using natural samples in the future. In addition, the isolated As(III)-oxidizing bacteria primarily belonged to *Proteobacteria* (Supplementary Figure 1), and this may lead to an over-estimation of the relative abundance of *Proteobacteria* in identifying As(III)-oxidizers in nature. Indeed, various primer pair targeted *aioA* genes could not cover all the lineages of As(III)-oxidizers, and a gene diversity analysis may produce different taxonomic information. We think this bias could be settled with high-throughput strain isolation methods and the recently rising metagenome binning technology (to obtain the nearly complete genome without cultivation).

The conserved regions of the *aioA* gene used for primer design may be an important factor influencing the taxonomic resolution of the *aioA* clone sequences. The deg1F/deg1R primers targeted the sequence encoding the conserved [3Fe–4S]-binding motif of the AioA protein compared with the 1109F/1548R primers targeting the sequence for the molybdopterin center of the AioA protein and the M1-2F/M3-2R primers targeting these two conserved regions (Table 1). Combined with the taxonomic information obtained from the analyses involving these three primer pairs, the taxonomic resolution of the primer pairs targeting the molybdopterin center sequence was much better than that of the primers targeting the [3Fe–4S]-binding motif sequence.

### Differences in the *aioA* Genes From the Paddy Soil and Sediment Samples

Traditional molecular fingerprint methods, such as T-RFLP, DGGE, and clone library, have been used to investigate the distribution and diversity of *aioA* genes (Zhang J. et al., 2015; Han et al., 2017; Pipattanajaroenkul et al., 2021). These studies have reported that the community of As(III)-oxidizing bacteria in paddy soil was dominated by *Alphaproteobacteria*, *Betaproteobacteria*, and *Gammaproteobacteria* (Dong et al., 2014; Jia et al., 2014), most of which are affiliated with *Rhizobiales*, *Burkholderiales*, *Comamonadaceae*, *Phyllobacteriaceae*, *Bradyrhizobiaceae*, and *Methylobacteriaceae* (Zhang S. Y. et al., 2015). In this study, the bacterial diversity and compositions of the *aioA* gene in paddy soil and sediment were first explored using NGS. Compared with the sediment, the paddy soil samples were significantly enriched in the *aioA* gene affiliated with the genus of *Devosia* (in the family of *Hyphomicrobiaceae*), *Kaistia* (in the family of *Rhizobiaceae*), and *Ralstonia* (in the family of *Burkholderiaceae*) (Figure 3). *Devosia* dominated in the community of the heterotrophic enrichment using As(III) as the only electron donor, suggesting its important role in As(III) oxidation in soil (Sultana et al., 2012). *Kaistia* is typically a rhizospheric genera, and the *aioA* gene has been identified in the genome of *Kaistia* sp. SCN 65-12 (Pal and Sengupta, 2021). *Ralstonia* sp. 22 was isolated from mine soil and shown to be capable of As(III) oxidation (Lieutaud et al., 2010). In addition, *Ralstonia* sp. b3 was the predominant species of an autotrophic arsenic(III)-oxidizing population in reactors (Battaglia-Brunet et al., 2002). Additionally, the relative abundance of *Curvibacter* (in the family of *Comamonadaceae*) was much higher in sediment than that in paddy soil samples (Figure 3). In a study of metagenomic insights into microbial arsenic metabolism in shallow groundwater, *aioA* genes primarily contributed to *Curvibacter* (Wang et al., 2020). In addition, the ferrous-oxidizing *Curvibacter* sp. CD03 has been retrieved from alluvial sediment (Hassan et al., 2016), indicating the bacteria in the genus *Curvibacter* have potential for As(III) and Fe(II) oxidation. This simultaneously suggests that it could be utilized as a bioremediation agent for reducing As toxicity and immobilization in sediments.

In this study, the majority of abundant OTUs in the paddy soil was placed to uncultured *aioA* sequences reported previously that are not affiliated with any cultured isolates. However, the OTUs from the sediment samples always clustered with reference *aioA* sequences of As(III)-oxidizing strains, such as *Thiobacillus* sp. SCN 64-35, *Comamonadaceae* bacterium SCN 68-20, and *Curvibacter* sp. GWA2 64 110 (Supplementary Figure 2). Although very few As(III) oxidizers have been successfully isolated from the paddy soil environment (only *Paracoccus* sp. SY and *Acidovorax* sp. ST3 have been reported) (Zhang J. et al., 2015; Zhang et al., 2017), the predicted AioA protein sequences of some OTUs from the paddy soil shared a high amino acid identity with well-known As(III) oxidizers, such as *Rhizobium* sp. NT-26, *H. arsenicoxydans*, and *Agrobacterium* sp. C13 (Supplementary Figure 2). These microbes have been shown to contribute significantly to the oxidation of As(III) under both aerobic and anaerobic conditions. The above findings indicated that the As(III)-oxidizing community structure and composition in the paddy soil and sediment environments were remarkably different. Therefore, additional efforts should be dedicated toward delineation of the diversity and distribution pattern of the As(III) oxidizers from diverse environments globally, and this would involve high-throughput parallel sequencing of a large number of samples.

### Environmental Factors Driving the Diversity of As(III)-Oxidizing Bacteria in Natural Environments

Microbial As(III) oxidation contributes to efficient As immobilization in environments. Thus, an understanding of the environmental factors that influence *aioA* genes is important for the bioremediation of arsenic-contaminated environments in the future. We found that the Chao1 and Faith PD diversity indices of the *aioA* genes were significantly higher in paddy soil than in sediment (Supplementary Table 5). In a previous study, a redundancy analysis indicated that soil pH, available Ca and P, and As(V) concentration were key factors driving diverse compositions in the *aioA* gene community, and As(V) amendment elevated *aioA* gene diversity in the rhizosphere of the As-hyperaccumulator *Pteris vittata* (Han et al., 2017). Zhang S. Y. et al. (2015) also found pH, SO_4_^2–^-S, and the total C/N ratio significantly explained the variation in the microbial community compositions based on *aioA* genes. However, the effects of arsenic species on the As(III)-oxidizing communities have been less reported. To explore how environmental parameters affected the diversity of *aioA* genes, an RF analysis was conducted. The results showed that dissolved As(III) had the highest relative influence on the Chao1 index of the *aioA* genes (Figure 4). In arsenic contaminated soils, phosphate-extractable As was one of the most important factors in shaping the As transformation functional genes (Gu et al., 2017). Furthermore, As(III) was identified as the major environmental factor influencing the community and abundance of As(III)-oxidizing bacteria in groundwater (Pipattanajaroenkul et al., 2021). Normally, As fractionation in soil and sediment includes dissolved, poorly crystalline Fe (oxyhydr)oxide-bound (extracted by NH_4_^+^-oxalate) and Fe (oxyhydr)oxides-bound (extracted by KH_2_PO_4_ and ascorbic acid) forms that represent the different degrees of bioavailability (Liu et al., 2015). It has been shown that dissolved As(III) is easily utilized as electron donor by bacteria as Fe (oxyhydr)oxide-bound As(III) (Jeong et al., 2010). Thus, it is not strange that the diversity of the *aioA* genes were very highly influenced by dissolved As(III) than by PO_4_-As(III) and oxalate- As(III).

## Conclusion

In summary, in the current study, it was demonstrated that the newly developed primer pair, 1109F/1548R, was sufficiently long to capture genetic variation, allowing for the discrimination of phylogenetic groups of the *aioA* genes. The obtained data encourages further use of the *aioA* gene as a functional marker specific to As(III) oxidizers in environmental diversity surveys. The newly developed *aioA* primers tested in the current study will aid other researchers wishing to detect As(III) oxidases (or their expression) in environmental systems, especially for the identification of the As(III)-oxidizing community composition and diversity, which requires multiple-sample analysis and deep sequencing. Future studies should focus on evaluating the link between As speciation and concentrations and *aioA* gene diversity and abundances in the environment.

## Data Availability Statement

The datasets presented in this study can be found in online repositories. The names of the repository/repositories and accession number(s) can be found in the article/Supplementary Material.

## Author Contributions

MH and FL conceived and designed the experiments. MH and JQ performed most of the experiments. CY, HY, and LZ supervised the execution of the experiments. MH wrote the manuscript. All authors read and approved the final manuscript.

## Conflict of Interest

The authors declare that the research was conducted in the absence of any commercial or financial relationships that could be construed as a potential conflict of interest.

## Publisher’s Note

All claims expressed in this article are solely those of the authors and do not necessarily represent those of their affiliated organizations, or those of the publisher, the editors and the reviewers. Any product that may be evaluated in this article, or claim that may be made by its manufacturer, is not guaranteed or endorsed by the publisher.

## Supplementary Material

Supplementary Figure 1 Phylogenetics of the 67 *aioA* genes used for the primer design, and the hits (a pair matched the same sequence with a maximum of two mismatches and alignment length larger than 16 nt) of the two primer pair matches) of the three primer pairs to the 67 *aioA* gene sequences. The solid square represents a hit of the primer pair to the corresponding *aioA* gene sequences, whereas the hollow square represents the primer pair could not match to the *aioA* gene sequences. The order of the squares in each *aioA* sequence are shown as1109F/1548R, M1-2F/M3-2R, and deg1F/deg1R. Sequences of *arrA* from *Shewanella* sp. ANA and *Bacillus selenitireducens* MLS10 functioned as outgroups in building the phylogenetic tree.

Supplementary Figure 2 Neighbor-joining tree of the 20 most abundant OTUs in the library of the *aioA*-1109F/1548R. The average relative abundances (%) for the paddy soil and sediment samples are given to the right of each OTU. The scale bar indicates the sequence dissimilarity between the nodes.

Supplementary Table 1 Information of arsenite oxidase and the corresponding genes used in the primer design.

Supplementary Table 2 Geochemistry of the paddy soils and sediments used for the *aioA* genes high-throughput amplicon sequencing.

Supplementary Table 3 Barcoding primer sequences for the amplicon sequencing on the Illumina Hiseq platform.

Supplementary Table 4 The amplicon sequencing information and alpha diversity index of the *aioA* genes on the Illumina Hiseq platform using the two newly designed primer pairs.

Supplementary Table 5 The difference in α-diversity of the paddy soil and sediment samples in the libraries of 1109F/1548R.

Supplementary Table 6 Phylogenetics of the *aioA* OTUs (with relative abundance > 1%) with their closest relatives.
