# Supplementary figures and images for: New Arsenite Oxidase Gene (aioA) PCR Primers for Assessing Arsenite-Oxidizer Diversity in the Environment Using High-Throughput Sequencing
Source: Front Microbiol. 2021 Oct 6;12:691913. doi: 10.3389/fmicb.2021.691913 (PMC8527091; doi:10.3389/fmicb.2021.691913)

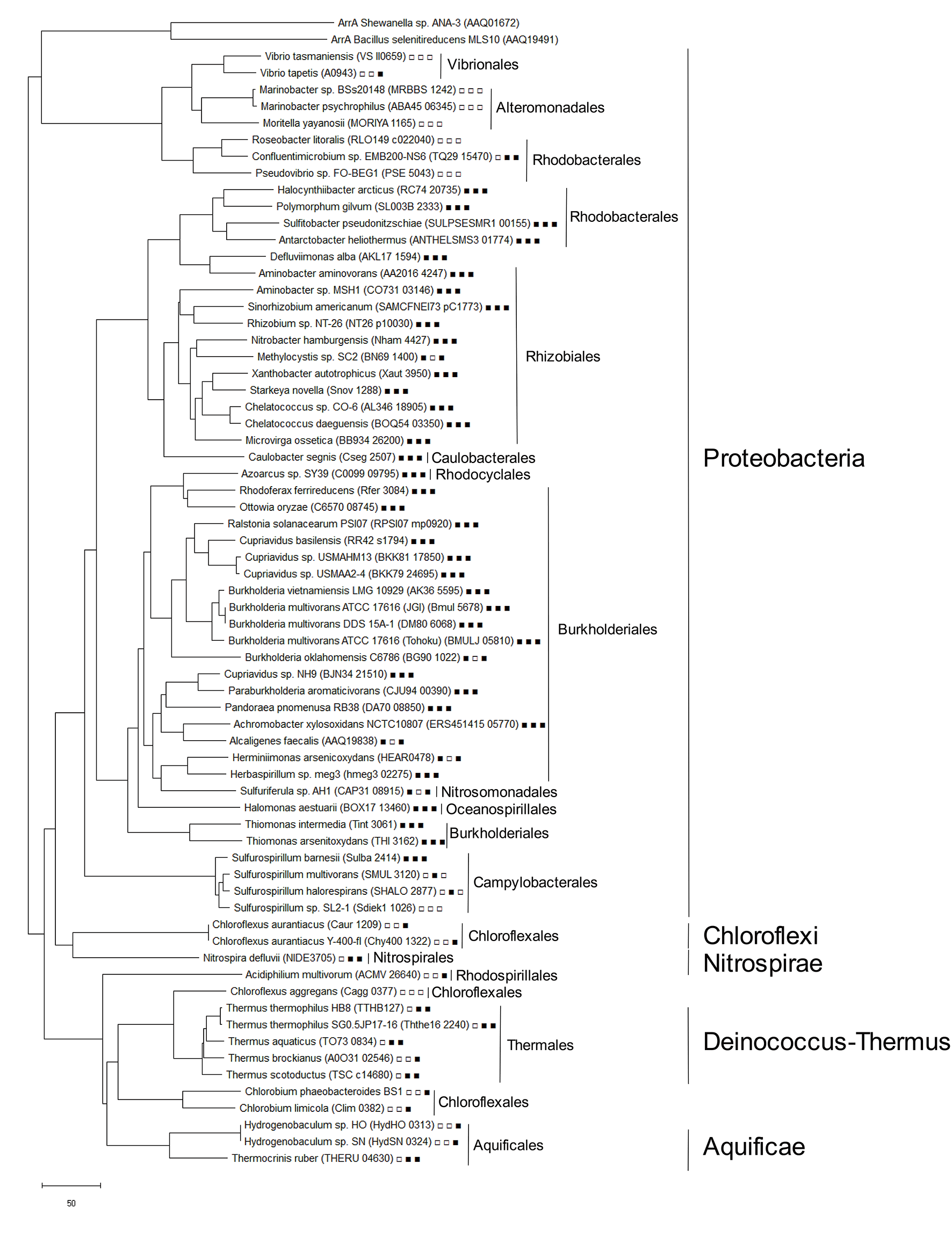

Supplement: Supplementary Figure 1 — Phylogenetics of the 67 aioA genes used for the primer design, and the hits (a pair matched the same sequence with a maximum of two mismatches and alignment length larger than 16 nt) of the two primer pair matches) of the three primer pairs to the 67 aioA gene sequences. The solid square represents a hit of the primer pair to the corresponding aioA gene sequences, whereas the hollow square represents the primer pair could not match to the aioA gene sequences. The order of the squares in each aioA sequence are shown as1109F/1548R, M1-2F/M3-2R, and deg1F/deg1R. Sequences of arrA from Shewanella sp. ANA and Bacillus selenitireducens MLS10 functioned as outgroups in building the phylogenetic tree. [file Image_1.TIF]

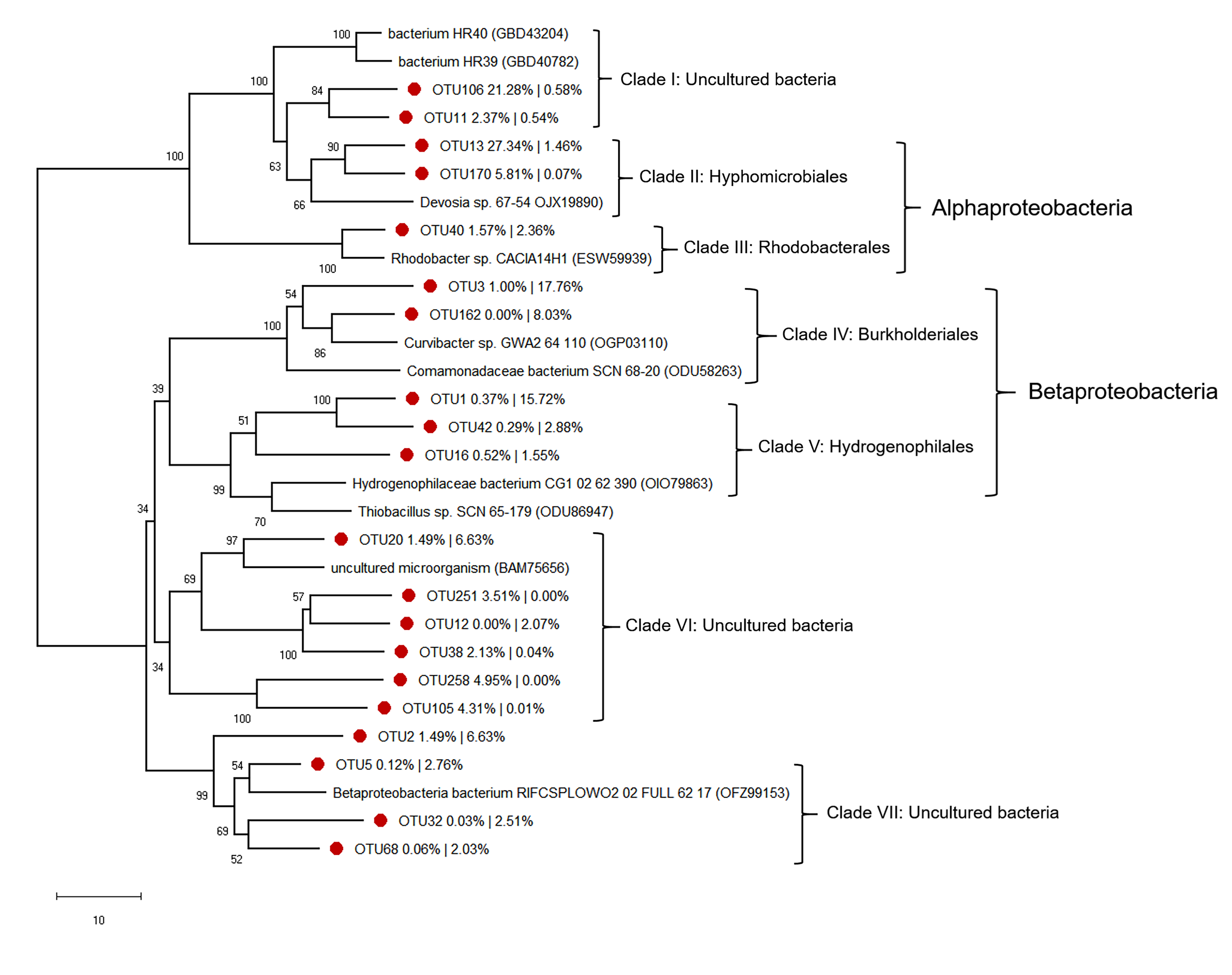

Supplement: Supplementary Figure 2 — Neighbor-joining tree of the 20 most abundant OTUs in the library of the aioA-1109F/1548R. The average relative abundances (%) for the paddy soil and sediment samples are given to the right of each OTU. The scale bar indicates the sequence dissimilarity between the nodes. [file Image_2.TIF]
