# Supplementary material for: New Arsenite Oxidase Gene (aioA) PCR Primers for Assessing Arsenite-Oxidizer Diversity in the Environment Using High-Throughput Sequencing
Source: Front Microbiol. 2021 Oct 6;12:691913. doi: 10.3389/fmicb.2021.691913 (PMC8527091; doi:10.3389/fmicb.2021.691913)
Supplement: Supplementary Table 1 — Information of arsenite oxidase and the corresponding genes used in the primer design. [file Table_1.DOCX]

**Supplementary Tables for**

**New Arsenite Oxidase Gene (*aioA*) PCR Primers for Assessing Arsenite-oxidizer Diversity in the Environment using High-Throughput Sequencing**

Min Hu^a,b*^, Fangbai Li^a,b^, Jiangtao Qiao^a,b^, Chaolei Yuan^c^, Huanyun Yu^a,b^, Li Zhuang^d^

^a^ Guangdong Key Laboratory of Integrated Agro-environmental Pollution Control and Management, Institute of Eco-environmental and Soil Sciences, Guangdong Academy of Sciences, Guangzhou 510650, China.

^b^ National-Regional Joint Engineering Research Center for Soil Pollution Control and Remediation in South China, Guangzhou 510650, China.

^c^ Ministry of Education Key Laboratory of Pollution Processes and Environmental Criteria, Tianjin Key Laboratory of Environmental Remediation and Pollution Control, College of Environmental Science and Engineering, Nankai University, Tianjin 300350, China.

^d^ School of Environment, Jinan University, Guangzhou 510632, China.

∗ *Correspondence to: Min Hu, Institute of Eco-environmental and Soil Sciences, Guangdong Academy of Sciences, Guangzhou 510650, China. E-mail:* [*humin@soil.gd.cn*](mailto:humin@soil.gd.cn)*.*

**Table S1**. Information of arsenite oxidase and the corresponding genes used in the primer design.

| **KEGG accession No.** | **Species** | **Phylogenetic position** | | | | | | **protein length** | **gene length** |
| --- | --- | --- | --- | --- | --- | --- | --- | --- | --- |
|  |  | **Kingdom** | **Phylum** | **Class** | **Order** | **Family** | **Genus** |  |  |
| hho:HydHO_0313 | Hydrogenobaculum sp. HO | Bacteria | Aquificae | Aquificae | Aquificales | Aquificaceae | Hydrogenobaculum | 873 | 2622 |
| hys:HydSN_0324 | Hydrogenobaculum sp. SN | Bacteria | Aquificae | Aquificae | Aquificales | Aquificaceae | Hydrogenobaculum | 873 | 2622 |
| trd:THERU_04630 | Thermocrinis ruber | Bacteria | Aquificae | Aquificae | Aquificales | Aquificaceae | Thermocrinis | 873 | 2622 |
| cpb:Cphamn1_2365 | Chlorobium phaeobacteroides | Bacteria | Chlorobi | Chlorobia | Chlorobiales | Chlorobiaceae | Chlorobium | 853 | 2562 |
| cli:Clim_0382 | Chlorobium limicola | Bacteria | Chlorobi | Chlorobia | Chlorobiales | Chlorobiaceae | Chlorobium | 854 | 2565 |
| cau:Caur_1209 | Chloroflexus aurantiacus | Bacteria | Chloroflexi | Chloroflexia | Chloroflexales | Chloroflexaceae | Chloroflexus | 836 | 2511 |
| chl:Chy400_1322 | Chloroflexus aurantiacus | Bacteria | Chloroflexi | Chloroflexia | Chloroflexales | Chloroflexaceae | Chloroflexus | 836 | 2511 |
| cag:Cagg_0377 | Chloroflexus aggregans | Bacteria | Chloroflexi | Chloroflexia | Chloroflexales | Chloroflexaceae | Chloroflexus | 861 | 2586 |
| ttj:TTHB127 | Thermus thermophilus | Bacteria | Deinococcus-Thermus | Deinococci | Thermales | Thermaceae | Thermus | 861 | 2586 |
| tts:Ththe16_2240 | Thermus thermophilus | Bacteria | Deinococcus-Thermus | Deinococci | Thermales | Thermaceae | Thermus | 861 | 2586 |
| tsc:TSC_c14680 | Thermus scotoductus | Bacteria | Deinococcus-Thermus | Deinococci | Thermales | Thermaceae | Thermus | 861 | 2586 |
| taq:TO73_0834 | Thermus aquaticus | Bacteria | Deinococcus-Thermus | Deinococci | Thermales | Thermaceae | Thermus | 861 | 2586 |
| tbc:A0O31_02546 | Thermus brockianus | Bacteria | Deinococcus-Thermus | Deinococci | Thermales | Thermaceae | Thermus | 861 | 2586 |
| nde:NIDE3705 | Nitrospira defluvii | Bacteria | Nitrospirae | Nitrospira | Nitrospirales | Nitrospiraceae | Nitrospira | 820 | 2463 |
| cse:Cseg_2507 | Caulobacter segnis | Bacteria | Proteobacteria | Alphaproteobacteria | Caulobacterales | Caulobacteraceae | Caulobacter | 822 | 2469 |
| nha:Nham_4427 | Nitrobacter hamburgensis | Bacteria | Proteobacteria | Alphaproteobacteria | Rhizobiales | Bradyrhizobiaceae | Nitrobacter | 820 | 2463 |
| chel:AL346_18905 | Chelatococcus sp. CO-6 | Bacteria | Proteobacteria | Alphaproteobacteria | Rhizobiales | Chelatococcaceae | Chelatococcus | 823 | 2472 |
| cdq:BOQ54_03350 | Chelatococcus daeguensis | Bacteria | Proteobacteria | Alphaproteobacteria | Rhizobiales | Chelatococcaceae | Chelatococcus | 823 | 2472 |
| moc:BB934_26200 | Microvirga ossetica | Bacteria | Proteobacteria | Alphaproteobacteria | Rhizobiales | Methylobacteriaceae | Microvirga | 874 | 2625 |
| msc:BN69_1400 | Methylocystis sp. SC2 | Bacteria | Proteobacteria | Alphaproteobacteria | Rhizobiales | Methylocystaceae | Methylocystis | 820 | 2463 |
| aak:AA2016_4247 | Aminobacter aminovorans | Bacteria | Proteobacteria | Alphaproteobacteria | Rhizobiales | Phyllobacteriaceae | Aminobacter | 822 | 2469 |
| amih:CO731_03146 | Aminobacter sp. MSH1 | Bacteria | Proteobacteria | Alphaproteobacteria | Rhizobiales | Phyllobacteriaceae | Aminobacter | 811 | 2436 |
| rht:NT26_p10030 | Rhizobium sp. NT-26 | Bacteria | Proteobacteria | Alphaproteobacteria | Rhizobiales | Rhizobiaceae | Rhizobium | 845 | 2538 |
| same:SAMCFNEI73_pC1773 | Sinorhizobium americanum | Bacteria | Proteobacteria | Alphaproteobacteria | Rhizobiales | Rhizobiaceae | Sinorhizobium | 874 | 2625 |
| sno:Snov_1288 | Starkeya novella | Bacteria | Proteobacteria | Alphaproteobacteria | Rhizobiales | Xanthobacteraceae | Starkeya | 820 | 2463 |
| xau:Xaut_3950 | Xanthobacter autotrophicus | Bacteria | Proteobacteria | Alphaproteobacteria | Rhizobiales | Xanthobacteraceae | Xanthobacter | 820 | 2463 |
| aht:ANTHELSMS3_01774 | Antarctobacter heliothermus | Bacteria | Proteobacteria | Alphaproteobacteria | Rhodobacterales | Rhodobacteraceae | Antarctobacter | 821 | 2466 |
| con:TQ29_15470 | Confluentimicrobium sp. EMB200-NS6 | Bacteria | Proteobacteria | Alphaproteobacteria | Rhodobacterales | Rhodobacteraceae | Confluentimicrobium | 891 | 2676 |
| daa:AKL17_1594 | Defluviimonas alba | Bacteria | Proteobacteria | Alphaproteobacteria | Rhodobacterales | Rhodobacteraceae | Defluviimonas | 819 | 2460 |
| hat:RC74_20735 | Halocynthiibacter arcticus | Bacteria | Proteobacteria | Alphaproteobacteria | Rhodobacterales | Rhodobacteraceae | Halocynthiibacter | 817 | 2454 |
| psf:PSE_5043 | Pseudovibrio sp. FO-BEG1 | Bacteria | Proteobacteria | Alphaproteobacteria | Rhodobacterales | Rhodobacteraceae | Pseudovibrio | 891 | 2676 |
| rli:RLO149_c022040 | Roseobacter litoralis | Bacteria | Proteobacteria | Alphaproteobacteria | Rhodobacterales | Rhodobacteraceae | Roseobacter | 891 | 2676 |
| spse:SULPSESMR1_00155 | Sulfitobacter pseudonitzschiae | Bacteria | Proteobacteria | Alphaproteobacteria | Rhodobacterales | Rhodobacteraceae | Sulfitobacter | 821 | 2466 |
| amv:ACMV_26640 | Acidiphilium multivorum | Bacteria | Proteobacteria | Alphaproteobacteria | Rhodospirillales | Acetobacteraceae | Acidiphilium | 861 | 2586 |
| pgv:SL003B_2333 | Polymorphum gilvum | Bacteria | Proteobacteria | Alphaproteobacteria | unclassified_Alphaproteobacteria | unclassified_Alphaproteobacteria | Polymorphum | 819 | 2460 |
| axx:ERS451415_05770 | Achromobacter xylosoxidans | Bacteria | Proteobacteria | Betaproteobacteria | Burkholderiales | Alcaligenaceae | Achromobacter | 827 | 2484 |
| ag:AAQ19838 | Alcaligenes faecalis | Bacteria | Proteobacteria | Betaproteobacteria | Burkholderiales | Alcaligenaceae | Alcaligenes | 826 | 2478 |
| boc:BG90_1022 | Burkholderia oklahomensis | Bacteria | Proteobacteria | Betaproteobacteria | Burkholderiales | Burkholderiaceae | Burkholderia | 826 | 2481 |
| bve:AK36_5595 | Burkholderia vietnamiensis | Bacteria | Proteobacteria | Betaproteobacteria | Burkholderiales | Burkholderiaceae | Burkholderia | 829 | 2490 |
| bmu:Bmul_5678 | Burkholderia multivorans | Bacteria | Proteobacteria | Betaproteobacteria | Burkholderiales | Burkholderiaceae | Burkholderia | 829 | 2490 |
| bmj:BMULJ_05810 | Burkholderia multivorans | Bacteria | Proteobacteria | Betaproteobacteria | Burkholderiales | Burkholderiaceae | Burkholderia | 829 | 2490 |
| bmk:DM80_6068 | Burkholderia multivorans | Bacteria | Proteobacteria | Betaproteobacteria | Burkholderiales | Burkholderiaceae | Burkholderia | 829 | 2490 |
| cbw:RR42_s1794 | Cupriavidus basilensis | Bacteria | Proteobacteria | Betaproteobacteria | Burkholderiales | Burkholderiaceae | Cupriavidus | 828 | 2487 |
| ccup:BKK81_17850 | Cupriavidus sp. USMAHM13 | Bacteria | Proteobacteria | Betaproteobacteria | Burkholderiales | Burkholderiaceae | Cupriavidus | 828 | 2487 |
| cuu:BKK79_24695 | Cupriavidus sp. USMAA2-4 | Bacteria | Proteobacteria | Betaproteobacteria | Burkholderiales | Burkholderiaceae | Cupriavidus | 828 | 2487 |
| cuh:BJN34_21510 | Cupriavidus sp. NH9 | Bacteria | Proteobacteria | Betaproteobacteria | Burkholderiales | Burkholderiaceae | Cupriavidus | 825 | 2478 |
| ppno:DA70_08850 | Pandoraea pnomenusa | Bacteria | Proteobacteria | Betaproteobacteria | Burkholderiales | Burkholderiaceae | Pandoraea | 825 | 2478 |
| parb:CJU94_00390 | Paraburkholderia aromaticivorans | Bacteria | Proteobacteria | Betaproteobacteria | Burkholderiales | Burkholderiaceae | Paraburkholderia | 825 | 2478 |
| rsl:RPSI07_mp0920 | Ralstonia solanacearum | Bacteria | Proteobacteria | Betaproteobacteria | Burkholderiales | Burkholderiaceae | Ralstonia | 828 | 2487 |
| otk:C6570_08745 | Ottowia sp. KADR8-3 | Bacteria | Proteobacteria | Betaproteobacteria | Burkholderiales | Comamonadaceae | Ottowia | 827 | 2484 |
| rfr:Rfer_3084 | Rhodoferax ferrireducens | Bacteria | Proteobacteria | Betaproteobacteria | Burkholderiales | Comamonadaceae | Rhodoferax | 827 | 2484 |
| hee:hmeg3_02275 | Herbaspirillum sp. meg3 | Bacteria | Proteobacteria | Betaproteobacteria | Burkholderiales | Oxalobacteraceae | Herbaspirillum | 826 | 2481 |
| har:HEAR0478 | Herminiimonas arsenicoxydans | Bacteria | Proteobacteria | Betaproteobacteria | Burkholderiales | Oxalobacteraceae | Herminiimonas | 826 | 2481 |
| tin:Tint_3061 | Thiomonas intermedia | Bacteria | Proteobacteria | Betaproteobacteria | Burkholderiales | unclassified_Burkholderiales | Thiomonas | 841 | 2526 |
| thi:THI_3162 | Thiomonas arsenitoxydans | Bacteria | Proteobacteria | Betaproteobacteria | Burkholderiales | unclassified_Burkholderiales | Thiomonas | 841 | 2526 |
| sulf:CAP31_08915 | Sulfuriferula sp. AH1 | Bacteria | Proteobacteria | Betaproteobacteria | Nitrosomonadales | Gallionellaceae | Sulfuriferula | 827 | 2484 |
| atw:C0099_09795 | Azoarcus sp. SY39 | Bacteria | Proteobacteria | Betaproteobacteria | Rhodocyclales | Zoogloeaceae | Azoarcus | 827 | 2484 |
| sba:Sulba_2414 | Sulfurospirillum barnesii | Bacteria | Proteobacteria | Epsilonproteobacteria | Campylobacterales | Campylobacteraceae | Sulfurospirillum | 823 | 2472 |
| smul:SMUL_3120 | Sulfurospirillum multivorans | Bacteria | Proteobacteria | Epsilonproteobacteria | Campylobacterales | Campylobacteraceae | Sulfurospirillum | 823 | 2472 |
| shal:SHALO_2877 | Sulfurospirillum halorespirans | Bacteria | Proteobacteria | Epsilonproteobacteria | Campylobacterales | Campylobacteraceae | Sulfurospirillum | 823 | 2472 |
| suls:Sdiek1_1026 | Sulfurospirillum sp. SL2-1 | Bacteria | Proteobacteria | Epsilonproteobacteria | Campylobacterales | Campylobacteraceae | Sulfurospirillum | 678 | 2037 |
| mbs:MRBBS_1242 | Marinobacter sp. BSs20148 | Bacteria | Proteobacteria | Gammaproteobacteria | Alteromonadales | Alteromonadaceae | Marinobacter | 900 | 2703 |
| mpq:ABA45_06345 | Marinobacter psychrophilus | Bacteria | Proteobacteria | Gammaproteobacteria | Alteromonadales | Alteromonadaceae | Marinobacter | 900 | 2703 |
| mya:MORIYA_1165 | Moritella yayanosii | Bacteria | Proteobacteria | Gammaproteobacteria | Alteromonadales | Moritellaceae | Moritella | 898 | 2697 |
| hsi:BOX17_13460 | Halomonas aestuarii | Bacteria | Proteobacteria | Gammaproteobacteria | Oceanospirillales | Halomonadaceae | Halomonas | 825 | 2478 |
| vsp:VS_II0659 | Vibrio tasmaniensis | Bacteria | Proteobacteria | Gammaproteobacteria | Vibrionales | Vibrionaceae | Vibrio | 914 | 2745 |
| vta:A0943 | Vibrio tapetis | Bacteria | Proteobacteria | Gammaproteobacteria | Vibrionales | Vibrionaceae | Vibrio | 893 | 2682 |

**Table S2.** Geochemistry of the paddy soils and sediments used for the *aioA* genes high-throughput amplicon sequencing.

| **Sample ID** | **Biotype** | **Geographical coordinates** | **pH** | **CEC(cmol/kg(+))** | **Organic matter (g kg-1)** | **As species (mg kg ^-1^)** | | | | | | |
| --- | --- | --- | --- | --- | --- | --- | --- | --- | --- | --- | --- | --- |
|  |  |  |  |  |  | **Total** | **Dissolved As(III)** | **Dissolved As(V)** | **PO_4_- As(III) ^1^** | **PO_4_--As(V) ^1^** | **Oxalate-As(III) ^2^** | **Oxalate-As(V) ^2^** |
| P1 | paddy soil | 23.617376 N 116.871372 E | 6.62 | 15.65 | 25.68 | 73.49 | 0.23 | 0.04 | 1.59 | 10.92 | 0.62 | 4.42 |
| P2 | paddy soil | 23.618472 N 116.876013 E | 6.44 | 15.86 | 31.55 | 51.72 | 0.13 | 0.06 | 0.85 | 5.05 | 0.56 | 2.38 |
| P3 | paddy soil | 23.614887 N 116.87554 E | 6.55 | 13.13 | 25.47 | 94.42 | 0.09 | 0.03 | 0.75 | 3.25 | 0.62 | 2.92 |
| P4 | paddy soil | 23.623108 N 116.858058 E | 6.63 | 15.17 | 28.78 | 54.18 | 0.19 | 0.10 | 2.83 | 6.45 | 1.08 | 4.20 |
| P5 | paddy soil | 23.6184547 N 116.938463 E | 6.75 | 12.02 | 27.8 | 37.19 | 0.07 | 0.04 | 0.48 | 4.75 | 0.87 | 3.69 |
| S1 | sediment | 23.6194778 N 116.883045 E | 6.49 | 18.76 | 35.33 | 40.44 | 0.11 | 0.08 | 0.83 | 3.42 | 0.72 | 4.39 |
| S2 | sediment | 23.616376 N 116.874372 E | 6.42 | 14.07 | 22.09 | 36.18 | 1.34 | 0.89 | 10.90 | 34.48 | 2.43 | 15.58 |
| S3 | sediment | 23.618572 N 116.886013 E | 6.48 | 17.56 | 36.74 | 64.22 | 0.36 | 0.05 | 4.06 | 34.19 | 1.20 | 13.93 |
| S4 | sediment | 23.613887 N 116.87654 E | 6.23 | 16.2 | 24.19 | 89.45 | 0.48 | 0.06 | 3.28 | 16.78 | 1.05 | 8.82 |
| S5 | sediment | 23.623118 N 116.858057 E | 6.15 | 12.96 | 17.19 | 55.7 | 0.07 | 0.02 | 1.60 | 12.84 | 0.89 | 6.50 |

^1^ Phosphate-extractable As(III) and As(V).

^2^ Oxalate-extractable As(III) and As(V).

**Table S3**. Barcoding primer sequences for the amplicon sequencing on the Illumina Hiseq platform.

|  | **Primer ID** | **Sequence (5'-3')** |
| --- | --- | --- |
| Reverse primer | aioA-1109F2-HS | AATGATACGGCGACCACCGAGATCTACACTATGGTAATTGTATCTGGGGBAAYRACAAYTA |
|  |  |  |
| Forward primer |  |  |
|  | aioA-1548R1-HS1 | CAAGCAGAAGACGGCATACGAGATTCCCTTGTCTCCAGTCAGTCAGCCTTCATBGASGTSAGRTTCAT |
|  | aioA-1548R1-HS2 | CAAGCAGAAGACGGCATACGAGATACGAGACTGATTAGTCAGTCAGCCTTCATBGASGTSAGRTTCAT |
|  | aioA-1548R1-HS3 | CAAGCAGAAGACGGCATACGAGATGCTGTACGGATTAGTCAGTCAGCCTTCATBGASGTSAGRTTCAT |
|  | aioA-1548R1-HS4 | CAAGCAGAAGACGGCATACGAGATATCACCAGGTGTAGTCAGTCAGCCTTCATBGASGTSAGRTTCAT |
|  | aioA-1548R1-HS5 | CAAGCAGAAGACGGCATACGAGATTGGTCAACGATAAGTCAGTCAGCCTTCATBGASGTSAGRTTCAT |
|  | aioA-1548R1-HS6 | CAAGCAGAAGACGGCATACGAGATATCGCACAGTAAAGTCAGTCAGCCTTCATBGASGTSAGRTTCAT |
|  | aioA-1548R1-HS7 | CAAGCAGAAGACGGCATACGAGATGTCGTGTAGCCTAGTCAGTCAGCCTTCATBGASGTSAGRTTCAT |
|  | aioA-1548R1-HS8 | CAAGCAGAAGACGGCATACGAGATAGCGGAGGTTAGAGTCAGTCAGCCTTCATBGASGTSAGRTTCAT |
|  | aioA-1548R1-HS9 | CAAGCAGAAGACGGCATACGAGATATCCTTTGGTTCAGTCAGTCAGCCTTCATBGASGTSAGRTTCAT |
|  | aioA-1548R1-HS10 | CAAGCAGAAGACGGCATACGAGATTACAGCGCATACAGTCAGTCAGCCTTCATBGASGTSAGRTTCAT |
|  | aioA-1548R1-HS11 | CAAGCAGAAGACGGCATACGAGATACCGGTATGTACAGTCAGTCAGCCTTCATBGASGTSAGRTTCAT |
|  | aioA-1548R1-HS12 | CAAGCAGAAGACGGCATACGAGATAATTGTGTCGGAAGTCAGTCAGCCTTCATBGASGTSAGRTTCAT |
|  | aioA-1548R1-HS13 | CAAGCAGAAGACGGCATACGAGATTGCATACACTGGAGTCAGTCAGCCTTCATBGASGTSAGRTTCAT |
|  | aioA-1548R1-HS14 | CAAGCAGAAGACGGCATACGAGATAGTCGAACGAGGAGTCAGTCAGCCTTCATBGASGTSAGRTTCAT |
|  | aioA-1548R1-HS15 | CAAGCAGAAGACGGCATACGAGATACCAGTGACTCAAGTCAGTCAGCCTTCATBGASGTSAGRTTCAT |
|  | aioA-1548R1-HS16 | CAAGCAGAAGACGGCATACGAGATGAATACCAAGTCAGTCAGTCAGCCTTCATBGASGTSAGRTTCAT |
|  | aioA-1548R1-HS17 | CAAGCAGAAGACGGCATACGAGATGTAGATCGTGTAAGTCAGTCAGCCTTCATBGASGTSAGRTTCAT |
|  | aioA-1548R1-HS18 | CAAGCAGAAGACGGCATACGAGATTAACGTGTGTGCAGTCAGTCAGCCTTCATBGASGTSAGRTTCAT |
|  | aioA-1548R1-HS19 | CAAGCAGAAGACGGCATACGAGATCATTATGGCGTGAGTCAGTCAGCCTTCATBGASGTSAGRTTCAT |
|  | aioA-1548R1-HS20 | CAAGCAGAAGACGGCATACGAGATCCAATACGCCTGAGTCAGTCAGCCTTCATBGASGTSAGRTTCAT |
|  | aioA-1548R1-HS21 | CAAGCAGAAGACGGCATACGAGATGATCTGCGATCCAGTCAGTCAGCCTTCATBGASGTSAGRTTCAT |
|  | aioA-1548R1-HS22 | CAAGCAGAAGACGGCATACGAGATCAGCTCATCAGCAGTCAGTCAGCCTTCATBGASGTSAGRTTCAT |
|  | aioA-1548R1-HS23 | CAAGCAGAAGACGGCATACGAGATCAAACAACAGCTAGTCAGTCAGCCTTCATBGASGTSAGRTTCAT |
|  | aioA-1548R1-HS24 | CAAGCAGAAGACGGCATACGAGATGCAACACCATCCAGTCAGTCAGCCTTCATBGASGTSAGRTTCAT |
|  | aioA-1548R1-HS25 | CAAGCAGAAGACGGCATACGAGATGCGATATATCGCAGTCAGTCAGCCTTCATBGASGTSAGRTTCAT |
|  | aioA-1548R1-HS26 | CAAGCAGAAGACGGCATACGAGATCGAGCAATCCTAAGTCAGTCAGCCTTCATBGASGTSAGRTTCAT |
|  | aioA-1548R1-HS27 | CAAGCAGAAGACGGCATACGAGATAGTCGTGCACATAGTCAGTCAGCCTTCATBGASGTSAGRTTCAT |
|  | aioA-1548R1-HS28 | CAAGCAGAAGACGGCATACGAGATGTATCTGCGCGTAGTCAGTCAGCCTTCATBGASGTSAGRTTCAT |
|  | aioA-1548R1-HS29 | CAAGCAGAAGACGGCATACGAGATCGAGGGAAAGTCAGTCAGTCAGCCTTCATBGASGTSAGRTTCAT |
|  | aioA-1548R1-HS30 | CAAGCAGAAGACGGCATACGAGATCAAATTCGGGATAGTCAGTCAGCCTTCATBGASGTSAGRTTCAT |
|  | aioA-1548R1-HS31 | CAAGCAGAAGACGGCATACGAGATAGATTGACCAACAGTCAGTCAGCCTTCATBGASGTSAGRTTCAT |
|  | aioA-1548R1-HS32 | CAAGCAGAAGACGGCATACGAGATAGTTACGAGCTAAGTCAGTCAGCCTTCATBGASGTSAGRTTCAT |
|  | aioA-1548R1-HS33 | CAAGCAGAAGACGGCATACGAGATGCATATGCACTGAGTCAGTCAGCCTTCATBGASGTSAGRTTCAT |
|  | aioA-1548R1-HS34 | CAAGCAGAAGACGGCATACGAGATCAACTCCCGTGAAGTCAGTCAGCCTTCATBGASGTSAGRTTCAT |
|  | aioA-1548R1-HS35 | CAAGCAGAAGACGGCATACGAGATTTGCGTTAGCAGAGTCAGTCAGCCTTCATBGASGTSAGRTTCAT |
|  | aioA-1548R1-HS36 | CAAGCAGAAGACGGCATACGAGATTACGAGCCCTAAAGTCAGTCAGCCTTCATBGASGTSAGRTTCAT |
|  | aioA-1548R1-HS37 | CAAGCAGAAGACGGCATACGAGATCACTACGCTAGAAGTCAGTCAGCCTTCATBGASGTSAGRTTCAT |
|  | aioA-1548R1-HS38 | CAAGCAGAAGACGGCATACGAGATTGCAGTCCTCGAAGTCAGTCAGCCTTCATBGASGTSAGRTTCAT |
|  | aioA-1548R1-HS39 | CAAGCAGAAGACGGCATACGAGATACCATAGCTCCGAGTCAGTCAGCCTTCATBGASGTSAGRTTCAT |
|  | aioA-1548R1-HS40 | CAAGCAGAAGACGGCATACGAGATTCGACATCTCTTAGTCAGTCAGCCTTCATBGASGTSAGRTTCAT |
|  | aioA-1548R1-HS41 | CAAGCAGAAGACGGCATACGAGATGAACACTTTGGAAGTCAGTCAGCCTTCATBGASGTSAGRTTCAT |
|  | aioA-1548R1-HS42 | CAAGCAGAAGACGGCATACGAGATGAGCCATCTGTAAGTCAGTCAGCCTTCATBGASGTSAGRTTCAT |
|  | aioA-1548R1-HS43 | CAAGCAGAAGACGGCATACGAGATTTGGGTACACGTAGTCAGTCAGCCTTCATBGASGTSAGRTTCAT |
|  | aioA-1548R1-HS44 | CAAGCAGAAGACGGCATACGAGATAAGGCGCTCCTTAGTCAGTCAGCCTTCATBGASGTSAGRTTCAT |
|  | aioA-1548R1-HS45 | CAAGCAGAAGACGGCATACGAGATTAATACGGATCGAGTCAGTCAGCCTTCATBGASGTSAGRTTCAT |
|  | aioA-1548R1-HS46 | CAAGCAGAAGACGGCATACGAGATTCGGAATTAGACAGTCAGTCAGCCTTCATBGASGTSAGRTTCAT |
|  | aioA-1548R1-HS47 | CAAGCAGAAGACGGCATACGAGATTGTGAATTCGGAAGTCAGTCAGCCTTCATBGASGTSAGRTTCAT |
|  | aioA-1548R1-HS48 | CAAGCAGAAGACGGCATACGAGATCATTCGTGGCGTAGTCAGTCAGCCTTCATBGASGTSAGRTTCAT |

**Table S4**. The amplicon sequencing information and alpha diversity index of the *aioA* genes on the Illumina Hiseq platform using the two newly designed primer pairs.

| **Samples** |  | **OTU status^1^** | | **α-diveristy index** | | | | | |
| --- | --- | --- | --- | --- | --- | --- | --- | --- | --- |
|  | ***aioA* sequences^2^** | **Total OTUs** | **OTUs after re-subsampling^3^** | **Chao1** | **Shannon** | **Simpson** | **Ace** | **Faith PD** | **Goods coverage** |
| P1 | 10,762 | 87 | 79 | 95.50 | 2.83 | 0.76 | 101.44 | 10.55 | 0.9978649 |
| P2 | 10,373 | 138 | 123 | 144.43 | 4.263 | 0.89 | 144.40 | 13.84 | 0.9975738 |
| P3 | 17,928 | 110 | 89 | 136.25 | 2.90 | 0.71 | 125.11 | 11.97 | 0.9972826 |
| P4 | 11,694 | 120 | 111 | 132.67 | 3.76 | 0.85 | 140.21 | 13.19 | 0.9974767 |
| P5 | 12,093 | 120 | 111 | 136.00 | 3.59 | 0.84 | 133.89 | 13.59 | 0.9974767 |
| S1 | 11,794 | 84 | 77 | 119.17 | 3.67 | 0.87 | 109.34 | 10.65 | 0.9977679 |
| S2 | 28,656 | 74 | 56 | 69.13 | 2.70 | 0.78 | 73.96 | 8.88 | 0.9985443 |
| S3 | 12,911 | 108 | 91 | 106.55 | 3.58 | 0.82 | 110.56 | 11.87 | 0.9981561 |
| S4 | 22,108 | 101 | 71 | 101.01 | 2.71 | 0.73 | 103.31 | 9.98 | 0.997962 |
| S5 | 12,968 | 138 | 112 | 127.33 | 3.28 | 0.76 | 129.96 | 13.10 | 0.9976708 |

^1^ OTU were clustered at 90% sequence identity.

^2^ The *aioA* gene sequences were verified by BLASTX similarity search against NCBI-nr database with e-value < e-10 and alignment length > 150 nt.

^3^ Each sample was randomly subsampling to 10,313 sequences.

**Table S5**. The difference in α-diversity of the paddy soil and sediment samples in the libraries of 1109F/1548R ^1^.

|  | **Paddy soil** | **Sediment** | **H** | ***p*-value** | ***q*-value** |
| --- | --- | --- | --- | --- | --- |
| Observed OTUs | 102.6±18.022 | 81.4±21.22 | 1.855 | 0.173 | 0.173 |
| Chao1 | 128.969±19.205 | 104.634±22.384 | 3.153 | 0.076 | 0.076 |
| Shannon | 3.468±0.605 | 3.19±0.465 | 1.320 | 0.251 | 0.251 |
| Simpson | 0.812±0.073 | 0.792±0.056 | 0.273 | 0.602 | 0.602 |
| Faith PD | 12.627±1.364 | 10.896±1.639 | 3.153 | 0.076 | 0.076 |

^1^ Kruskal-wallis test was used to identified the difference of α-diversity of paddy soil and sediment samples.

**Table S6.** Phylogenetics of *aioA* OTUs (with relative abundance > 1 %) with their closest relatives.

| **Clade** | ***aioA* OTUs** | **Closest relatives** | **Identity (%)** | **E value** | **Bit score** | **Coverage (%)** | **Affiliation** |
| --- | --- | --- | --- | --- | --- | --- | --- |
| II | OTU13 | *Devosia* sp. 67-54 (OJX19890) | 83.10 | 1.03E-82 | 251 | 100 | Hyphomicrobiales/Alphaproteobacteria |
|  | OTU170 | *Devosia* sp. 67-54 (OJX19890) | 82.39 | 3.19E-82 | 249 | 100 |  |
| III | OTU40 | *Rhodobacter* sp. CACIA14H1 (ESW59939) | 93.00 | 1.54E-93 | 279 | 100 | Rhodobacterales/Alphaproteobacteria |
| IV | OTU3 | *Comamonadaceae* bacterium SCN 68-20 (ODU58263) | 82.39 | 1.16E-84 | 256 | 100 | /Burkholderiales/Betaproteobacteria |
|  | OTU162 | *Curvibacter* sp. GWA2_64_110 (OGP03110) | 89.44 | 2E-91 | 274 | 100 |  |
|  | OTU2 | *Thiobacillus* sp. SCN 65-179 (ODU86947) | 69.93 | 1.76E-70 | 218 | 100 | Nitrosomonadales/Betaproteobacteria |
| V | OTU1 | *Hydrogenophilaceae* bacterium CG1_02_62_390 (OIO79863) | 82.39 | 1.24E-82 | 251 | 99 | Hydrogenophilales/Proteobacteria |
|  | OTU42 | *Hydrogenophilaceae* bacterium CG1_02_62_390 (OIO79863) | 80.28 | 6.7E-79 | 241 | 99 |  |
|  | OTU16 | *Hydrogenophilaceae* bacterium CG1_02_62_390 (OIO79863) | 76.06 | 9.28E-78 | 238 | 99 |  |
| I | OTU11 | Bacterium HR39 GBD40782) | 83.22 | 5.8E-83 | 252 | 100 | Uncultured bacteria |
|  | OTU106 | Bacterium HR40 (GBD43204) | 81.12 | 4.78E-81 | 247 | 100 |  |
| VII | OTU5 | Betaproteobacteria bacterium RIFCSPLOWO2_02_FULL_62_17 (OFZ99153) | 88.37 | 1.63E-79 | 242 | 91 | Uncultured bacteria |
|  | OTU32 | Betaproteobacteria bacterium RIFCSPLOWO2_02_FULL_62_17 (OFZ99153) | 86.29 | 9.37E-74 | 227 | 87 |  |
|  | OTU68 | Betaproteobacteria bacterium RIFCSPLOWO2_02_FULL_62_17 (OFZ99153) | 85.27 | 2.71E-73 | 226 | 91 |  |
| VI | OTU20 | Uncultured microorganism (BAM75656) | 86.53 | 7.75E-92 | 265 | 100 | Uncultured bacteria |
|  | OTU258 | Uncultured microorganism (BAM75656) | 70.92 | 1.53E-73 | 218 | 100 |  |
|  | OTU105 | Uncultured microorganism (BAM75656) | 72.86 | 1.97E-76 | 226 | 99 |  |
|  | OTU251 | Uncultured microorganism (BAM75656) | 75.89 | 2.76E-78 | 230 | 100 |  |
|  | OTU38 | Uncultured microorganism (BAM75656) | 75.89 | 4.25E-79 | 232 | 100 |  |
|  | OTU12 | Uncultured microorganism (BAM75656) | 75.18 | 2.85E-77 | 228 | 100 |  |
